# Defective Visceral Adipose Tissue Adaptation in Gestational Diabetes Mellitus

**DOI:** 10.1210/clinem/dgad699

**Published:** 2023-11-30

**Authors:** Colm J McElwain, Samprikta Manna, Andrea Musumeci, Isaac Sylvester, Chloé Rouchon, Anne Marie O'Callaghan, Mustafa Abdalla Bakhit Ebad, Fergus P McCarthy, Cathal M McCarthy

**Affiliations:** Department of Pharmacology and Therapeutics, Western Gateway Building, University College Cork, Cork T12XF62, Ireland; Department of Obstetrics and Gynaecology, Cork University Maternity Hospital, Cork T12DC4A, Ireland; Department of Pharmacology and Therapeutics, Western Gateway Building, University College Cork, Cork T12XF62, Ireland; Department of Pharmacology and Therapeutics, Western Gateway Building, University College Cork, Cork T12XF62, Ireland; Department of Pharmacology and Therapeutics, Western Gateway Building, University College Cork, Cork T12XF62, Ireland; Department of Pharmacology and Therapeutics, Western Gateway Building, University College Cork, Cork T12XF62, Ireland; Department of Pharmacology and Therapeutics, Western Gateway Building, University College Cork, Cork T12XF62, Ireland; Department of Obstetrics and Gynaecology, Cork University Maternity Hospital, Cork T12DC4A, Ireland; Department of Pharmacology and Therapeutics, Western Gateway Building, University College Cork, Cork T12XF62, Ireland

**Keywords:** gestational diabetes mellitus (GDM), visceral adipose tissue (VAT), insulin resistance, insulin signaling, adipogenesis, endocrinology

## Abstract

**Context:**

Gestational diabetes mellitus (GDM) is a complex obstetric condition affecting localized glucose metabolism, resulting in systemic metabolic dysfunction.

**Objective:**

This cross-sectional study aimed to explore visceral adipose tissue (VAT) as an integral contributor to GDM, focusing on elucidating the specific contribution of obesity and GDM pathology to maternal outcomes.

**Methods:**

Fifty-six nulliparous pregnant women were recruited, including normal glucose tolerant (NGT) (*n* = 30) and GDM (*n* = 26) participants. Participants were subgrouped as nonobese (BMI <30 kg/m^2^) or obese (BMI ≥30 kg/m^2^). Metabolic markers in circulation, VAT, and placenta were determined. Morphological analysis of VAT and immunoblotting of the insulin signaling cascade were performed.

**Results:**

GDM participants demonstrated hyperinsulinemia and elevated homeostatic model assessment for insulin resistance (HOMA-IR) scores relative to NGT participants. The GDM-obese subgroup had significant VAT adipocyte hypoplasia relative to NGT-nonobese tissue. GDM-obese VAT had significantly lower insulin receptor substrate (IRS)-2 expression, with elevated ser312 phosphorylation of IRS-1, relative to NGT-nonobese. GDM-obese participants had significantly elevated circulating leptin levels and placental adipsin secretion, while GDM-nonobese participants had elevated circulating adipsin levels with reduced placental adiponectin secretion.

**Conclusion:**

These findings suggest that GDM-obese pregnancy is specifically characterized by inadequate VAT remodeling and dysfunctional molecular signaling, which contribute to insulin resistance and hinder metabolic health.

Insulin resistance is a well-defined physiological response to healthy pregnancy, providing sufficient glucose for maternal and fetal requirements (1). Gestational diabetes mellitus (GDM) is a serious obstetric complication that affects approximately 10% to 15% of pregnancies worldwide and is characterized by an insufficient insulin response to compensate for the insulin-resistant state of pregnancy (2, 3). Maternal body mass index (BMI) is a major contributory risk factor for GDM diagnosis with a 6.79-fold increased risk in obese women and 2.29-fold increased risk in overweight women (4).

Adipose tissue plays a central role in regulating systemic glucose homeostasis via 2 primary mechanisms, the efficient storage of lipids and the modulation of adipokine secretion. These mechanisms are pivotal in maintaining numerous metabolic signaling cascades that prevent glucotoxicity and lipotoxic-mediated effects. Adipocyte dysfunction is a prominent driver of insulin resistance, with various mechanisms responsible for this pathological link. Insufficient fat sequestration in adipose tissue depots leads to elevated circulating levels of free fatty acids (FFAs) and triglycerides, which negatively affect insulin action on glucose metabolism (5). Increased adiposity can also alter the ability of adipocytes to function as endocrine cells and secrete appropriate biological mediators. This can lead to imbalances, including excessive release of monocyte chemoattractant protein 1 (MCP-1) with subsequent macrophage infiltration and meta-inflammation (6), or diminished secretion of adiponectin, resulting in reduced insulin sensitivity (7). Dysregulation of the intracellular adipocyte insulin signaling pathway can directly hinder glucose uptake. Previous studies of serum and skeletal muscle in women with GDM have found reduced insulin-stimulated glucose uptake, with decreased insulin receptor substrate (IRS)-1 expression, reduced tyrosine phosphorylation, and increased serine phosphorylation of the IRS-1 protein (8-10).

Compared with subcutaneous adipose tissue (SAT), visceral adipose tissue (VAT) morphology and functional output is associated with an increased risk of type 2 diabetes, hypertension, and dyslipidemia (11). Increased energy intake can often result in an insufficient number of mature adipocytes and a deficiency in VAT storage capacity (12). VAT physiologically responds to this deficiency via 2 mechanisms, initiating hypertrophy and/or hyperplasia. During healthy pregnancy, adipose tissue expands to support the nutritional needs of the growing fetus, reaching a peak toward the end of the second trimester, and deficient adipose tissue expansion is associated with insulin resistance, attributed to ectopic lipid deposition (13). An important mediator of physiological preadipocyte differentiation, and subsequent hyperplasia, is the action of insulin growth factor (IGF)-1, which is regulated locally by IGF-binding proteins (IGFBPs). These binding proteins have a dual role; they increase the half-life of IGF-1 locally and in the circulation and they block the potential binding of IGF-1 to both IGF-1 and insulin receptors (14). Furthermore, previous findings have shown that placenta-secreted hormones such as human placental lactogen (hPL), which has been proposed to drive insulin resistance in GDM (15), regulate lipolysis during healthy pregnancy, particularly during the third trimester (16).

The aim of this study was to identify molecular mechanisms in omental VAT which may contribute to GDM pathology, with a focus on defining those pathways which may be specifically driven by GDM diagnosis and/or obesity during pregnancy.

## Methods

### Study Design and Participant Recruitment

GDM-diagnosed (*n* = 26) and healthy normal glucose tolerant (NGT) control (*n* = 30) participants were recruited from Cork University Maternity Hospital (CUMH), Ireland between 2019 and 2022 as part of the COMRADES study, an observational, cross-sectional, cohort study of nulliparous singleton pregnancies with the aim of characterizing metabolic dysfunction in GDM pathology. The COMRADES study was conducted according to the guidelines laid down in the Declaration of Helsinki, and all the procedures were approved by the Clinical Research Ethics Committee of the Cork Teaching Hospitals (ECM4 [ff] 04/12/18). All participants provided informed written consent. Inclusion criteria for all study participants included women aged between 18 and 50 years, nulliparous, singleton pregnancy as determined by ultrasound scan, and indicated for an elective cesarean delivery. As per national Health Service Executive (HSE) guidelines, selective screening for GDM was carried out on identified at-risk pregnant women between 24 and 28 weeks gestation using a fasting blood glucose measurement. A diagnosis of GDM was confirmed if fasting glycemic levels were above 5.1 mmol/L.

Healthy NGT participants were defined as pregnant women who had not been diagnosed with GDM, as per the above guidelines, and did not present with any other diagnosed comorbidities. Exclusion criteria included women who had a multiple pregnancy, were multiparous, had already started to labor, who were outside the age range of 18 to 50 years of age, or who had an emergency cesarean delivery. Exclusion criteria included women with known pregnancy-related or other health-related conditions, namely, pre-pregnancy essential hypertension, known liver or renal diseases, known HIV, hepatitis B or hepatitis C positivity; known systemic lupus erythematous; known antiphospholipid syndrome, and known or suspected fetal anomalies.

### Sample Collection

Fasting blood samples were collected from study participants before surgery on the day of elective cesarean delivery. Blood samples were collected in EDTA vacutainer tubes for plasma collection and vacutainer serum tubes for serum collection. Samples were centrifuged at 2400*g* for 10 minutes at 4 °C before plasma and serum were collected. This method was performed according to a standardized laboratory protocol. Samples were then flash-frozen in liquid nitrogen and stored at −80 °C until analysis was performed. Visceral omentum tissue was collected from one site during cesarean delivery and processed within 30 minutes postsurgery. Placental tissue samples were obtained immediately after the cesarean delivery. Biological samples were flash-frozen and stored at −80 °C until analysis was performed. Additionally, tissue samples were taken for explant culture experiments or fixed in 10% formalin for histological analysis.

### Immunoassays

Plasma insulin, glucose, and IGF-1 were measured using the following kits: Insulin Quantikine ELISA kit (Bio-Techne DINS00, RRID:AB_3073852), Glucose assay (Abcam ab65333), and IGF-1 Quantikine ELISA kit (Bio-Techne DG100B, RRID:AB_2915951). Serum pregnancy associated plasma protein A (PAPPA) was measured by PAPPA Quantikine ELISA kit (Bio-Techne DPPA00, RRID:AB_3073853). To determine the extent of systemic insulin resistance in our study cohort, circulating glucose and insulin levels were used to calculate homeostatic model assessment for insulin resistance (HOMA-IR) values according to the formula: [fasting insulin (µU/L) × fasting glucose (nmol/L)/22.5]. Serum triglyceride levels were quantified using a triglyceride assay (Abcam 65336). Adiponectin, adipsin, leptin, and resistin were measured in plasma, omental, and placental explant culture supernatant using the LEGENDplex Human Metabolic Panel 1 (BioLegend 740212, RRID:AB_3073851). All assays were performed as per the manufacturer instructions.

### Morphological Analysis

VAT samples were fixed in 10% neutral buffered formalin (NBF) within 30 minutes of surgical biopsy and embedded in paraffin for histological investigation. Tissue sections (7 μm) of each sample were sectioned using a Leica RM2125 RTS microtome and mounted on SuperFrost glass slides (VWR 631-0446). Hematoxylin and eosin (H&E) morphological staining was performed on VAT samples using a standard protocol. All samples were blinded prior to image acquisition and analysis. Brightfield microscopy was used to visualize sections at 20× magnification. Images were imported into Image J—Fiji software for analysis. Adipocyte cell counting was performed on 5 images (at 20×) from different sections of each sample. Cell measurements were completed manually on 10 images per sample (2 images from each of 5 different sections). Adipocyte area (μm^2^) was calculated as the area of the ellipse from the perpendicular major (a) and minor (b) axes using the following equation: [Area = a × b × pi] (17). Picro Sirius Red (PSR) staining (Abcam ab150681) was used to quantify the extent of tissue fibrosis in VAT. Collagen fibers (red) in VAT were imaged using brightfield microscopy on 5 separate 20× images from different sections of each sample. Pericellular fibrosis was quantified in ImageJ software by converting the image to grayscale and segmenting the red-stained collagen using thresholding as per Image J protocol.

### Protein Isolation and Immunoblotting

VAT samples (50 mg ± 5 mg) were incubated in T-PER Tissue Protein Extraction Reagent (Thermo Scientific 78510) supplemented with phosphatase inhibitor (Roche, PhosSTOP) and protease inhibitor (Roche) and homogenized for 3× 20 seconds using a TissueLyser II (Qiagen). Isolated protein (30 μg) was separated at 100 V by SDS-PAGE on a 7.5% Tris-Glycine polyacrylamide gel and transferred to a methanol activated PVDF membrane (Amersham Hybond Sigma-Aldrich) at 400 mA on ice for 2 hours. Membranes were blocked with 5% bovine serum albumin in 0.1% TBS-T (tris-buffered saline with 0.1% Tween detergent) for 1 hour at room temperature and incubated overnight at 4 °C with the following primary antibodies: IGFBP3 1:1000 (R&D Systems AF675, RRID:AB_2123342); IGFBP4 1:3000 (R&D Systems AF804, RRID:AB_355615); IRS1 1:1000 (Cell Signaling #3407, RRID:AB_2127860); IRS2 1:1000 (Cell Signaling #4502, RRID:AB_2125774); phospho-IRS1(ser312) 1:1000 (Cell Signaling #2381, RRID:AB_330342); phospho-IRS1(ser323) 1:1000 (Cell Signaling #5610, RRID:AB_10695244); phospho-IRS1(ser616) 1:1000 (Cell Signaling #3203, RRID:AB_1031167); PDK1 1:1000 (Cell Signaling #3062, RRID:AB_2236832); PI3K 1:1000 (Cell Signaling #4249, RRID:AB_2165248); and GAPDH 1:1000 (Cell Signaling #97166, RRID:AB_2756824). After overnight incubation, membranes were washed and incubated at room temperature for 1 hour with secondary HRP-linked antibody, either anti-rabbit 1:2000 (Cell Signaling #7074, RRID:AB_2099233), anti-mouse 1:2000 (Santa Cruz 516102, RRID:AB_2687626), or anti-goat 1:1000 (R&D Systems HAF019, RRID:AB_573132). Membranes were washed in 0.1% TBS-T and developed using Pierce ECL Western Blotting Substrate (Thermo Scientific 32209) and LICOR Odyssey image analyzer. Densitometry was performed using LICOR Image Studio Lite Version 5.2. All densitometry values were normalized by relative GAPDH expression.

### Omental VAT and Placental Explant Culture

Tissue explants were prepared from omental VAT samples collected from both NGT and GDM participants. Samples of 100 mg ± 10 mg of omental tissue were cultured in 12-well plates, with 2 mL of culture media containing Medium 199 1× (Gibco 31150022), fetal bovine serum (heat-inactivated) 10% (Gibco 16140071), penicillin-streptomycin (10 000 U/mL) 1% (Gibco 15140122), and amphotericin B 0.25 µg/mL (Gibco 15290018). Tissue explants were also prepared from placental samples collected from both NGT and GDM participants. Following the removal of outer membrane and connective tissue, 200 mg ± 20 mg of placental tissue (combined from 3 regions of the placenta) was cultured in 12-well plates, containing 2 mL of culture media. Placental explant media consisted of RPMI-1640 1× (Gibco 21875034), fetal bovine serum (heat-inactivated) 10% (Gibco 16140071), penicillin-streptomycin (10 000 U/mL) 1% (Gibco 15140122), insulin 1 µg/mL (SAFC 91077C), hydrocortisone 0.1 µg/mL (Sigma H0888), retinol acetate 0.1 µg/mL (Sigma R0300000), and gentamicin 0.05 µg/mL (Sigma G1397). Tissue weight was recorded and used for normalization of all explant experimental analysis. Supernatants were subsequently stored at −80 °C for further analysis.

### Metabolic Experiments in Primary Human Preadipocyte

Primary preadipocytes at passage 4 were obtained from Caltag Medsystems (Zenbio OP-F-SL). These cells were a pool from nondiabetic, nonsmoker, nonpregnant female donors with a mean age of 37.7, a mean BMI of 46.5, and mixed ethnicity including African American, Caucasian, and Hispanic ethnic groups. Before culture, all plates were coated with 1.0 mL/25 cm^2^ poly-l-lysine (PLL) (Sigma P4832) to improve cell adherence and proliferation.

Preadipocytes were seeded at 5000 cells/cm^2^ in preadipocyte growth medium (PromoCell C-27410) and subcultured as per PromoCell protocols. Preadipocytes were differentiated with preadipocyte differentiation media (PromoCell C-27436). After 72 hours, differentiation media was aspirated, and adipocyte nutrition media (PromoCell C-27438) was added. Cells were maintained in nutrition media, with media change every 2 to 3 days, until adipocytes were fully differentiated (approximately 14 days).

To assess the effect of placental mediators on the preadipocyte differentiation, an adipogenesis assay was performed using the AdipoRed fluorescent assay (Lonza PT-7009). Primary preadipocyte cells were cultured as above in a 96-well plate with opaque walls (Corning 3610). Once preadipocytes reached confluency, differentiation medium was added ± 5% placental explant culture supernatant from either NGT or GDM participants. After a 72-hour culture period, media was aspirated and fluorescent staining with AdipoRed was performed as per the manufacturer's protocol. Briefly, the cell culture plate was cooled to room temperature and culture supernatant was carefully removed. Wells were washed with 200 µL PBS (Sigma P4417). Each well was then filled with 200 µL PBS and 5 µL AdipoRed reagent. After 10 minutes, fluorescence was measured with excitation at 485 nm and emission at 595 nm on a Tecan Spark spectrophotometer. Nine measurements were recorded in each well in a 3 × 3 pattern, covering the total well surface area. RIPA 1× (Millipore 20-188) was then added to each well for protein extraction for assay normalization. Cell protein lysate content was quantified using the Pierce BCA Protein assay kit (Thermo Scientific 23227).

To assess the effect of placental mediators on the depletion of intracellular triglycerides from terminally differentiated mature adipocytes, a lipid utilization assay was performed using the AdipoRed fluorescent assay reagent (Lonza PT-7009). Fully differentiated mature adipocytes were cultured in adipocyte nutrition media (PromoCell C-27438) with ± 5% treatment of placental explant culture supernatant from NGT and GDM participants for 24 hours. Fluorescent staining with AdipoRed was carried out described above. Fluorescence was measured with excitation at 485 nm and emission at 595 nm on a Tecan Spark spectrophotometer. Nine measurements were recorded in each well in a 3 × 3 pattern, covering the total well surface area. RIPA 1× (Millipore 20-188) was then added to each well for protein extraction and assay normalization. Cell protein lysate content was quantified using the Pierce BCA Protein assay kit (Thermo Scientific 23227).

To investigate the effect of placental mediators on glucose uptake in mature adipocytes, the Glucose Uptake-Glo assay (Promega J1342) was used. Fully differentiated mature adipocytes were cultured in adipocyte nutrition media (PromoCell C-27438) with ±5% treatment of placental explant culture supernatant from NGT and GDM participants for 24 hours. Glucose Uptake-Glo assay was performed as per the manufacturer's protocol. In short, media was removed, and cells were washed with 100 µL PBS (Sigma P4417). Then 50 µL of 1 mM 2-deoxyglucose was added to each well, briefly shaken, and incubated for 10 minutes at room temperature. Next 25 µL of stop buffer was added, followed by 25 µL of neutralization buffer. Finally, 100 µL of 2-deoxyglucose-6-phosphate detection reagent was added and the plate was agitated. Cells were incubated at room temperature for 2 hours and luminescence was measured on an Orion L microplate luminometer at 1 second/well.

### Statistics

Statistical analysis was performed using GraphPad Prism 9.5.1. Unpaired *t* test or Fisher exact test were used to compare clinical characteristics between NGT and GDM participants. Multiple linear regression analysis was used to evaluate the relationships between quantitative dependent variables and GDM diagnosis, adjusted for maternal age. Dependent variables were subgrouped by maternal BMI to investigate obesity effects (NGT-nonobese, NGT-obese, GDM-nonobese, GDM-obese). Obesity was categorized as a BMI ≥30 kg/m^2^ as recorded at their initial pregnancy clinic visit (8-12 weeks gestation). Within GDM participants, multiple linear regression was used to evaluate the effect on diagnosed treatment (diet-only, metformin, or insulin) on significant findings. *P* values <.05 were considered as statistically significant. The ROUT method was used to detect outliers in data sets. Data are represented as mean ± SD for samples sizes > *n* = 10 or median ± 95% CI for samples sizes ≤ *n* = 10.

## Results

### Baseline Characteristics of Study Participants

A total of 56 nulliparous pregnant women were included in this study, including NGT (*n* = 30) and GDM-diagnosed pregnancies (*n* = 26). Clinical characteristics were recorded from electronic health records for each individual and any differences between participant groups were assessed (Table 1). There were no significant differences observed between study groups for maternal age, fetal weight, placenta weight, fetal gender, or plasma glucose levels. GDM participants had a significantly higher BMI, lower gestational age at cesarean delivery, higher plasma insulin levels, and higher HOMA-IR scores relative to NGT participants.

**Table 1.** Clinical information for NGT (*n* = 30) and GDM (*n* = 26) participants

	NGT (*n* = 30)	GDM (*n* = 26)	*P* value
Maternal age, years	33.37 ± 4.16	35.23 ± 4.92	.13
Maternal BMI, kg/m^2^	26.23 ± 5.24	32.56 ± 5.87	.0001***
Gestational age, weeks	38.94 ± 0.49	38.38 ± 0.69	.0008***
Fetal weight, g	3435 ± 468.74	3559.62 ± 502.43	.34
Placenta weight, g	677.17 ± 157.69	723.6 ± 139.39	.31
Fetal sex	Male 40% (12)	Male 54% (14)	.42
	Female 60% (18)	Female 46% (12)	
GDM intervention	N/A	Diet—38% (10)	
		Metformin—27% (7)	
		Insulin—35% (9)	
Insulin, pmol/L	40.24 ± 14.55	123.85 ± 146.48	.01*
Glucose, mmol/L	5.98 ± 0.67	6.3 ± 0.27	.07
HOMA-IR	1.79 ± 0.72	4.59 ± 5.62	.03*

Unpaired *t* test was used to compare means between continuous variables. Fisher exact test was used to compare categorical outcomes. Where applicable, data are represented as mean ± SD. Significance levels are shown as: *P < .05, ***P < .001.

### Morphological Evidence of Adipocyte Hypoplasia in VAT in GDM-obese

**Figure 1.**
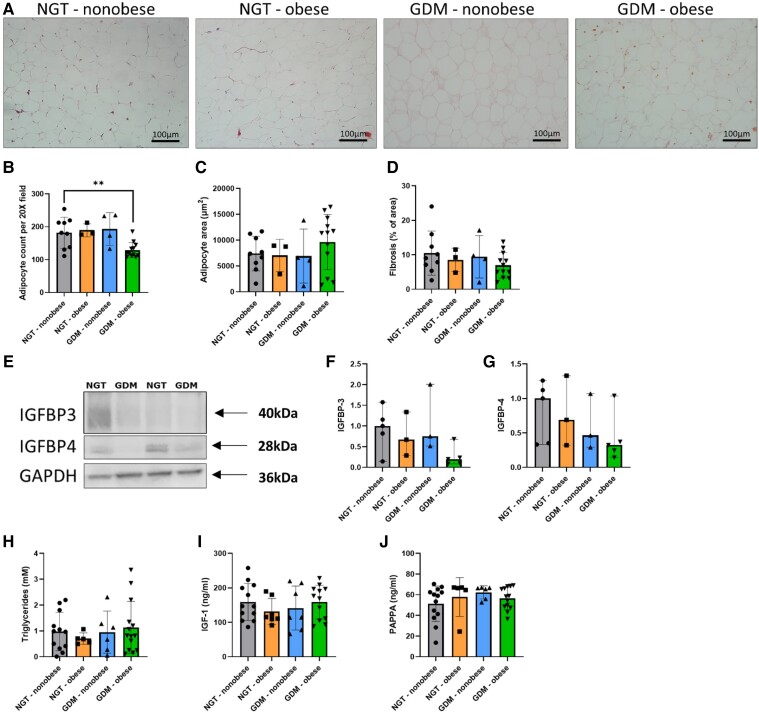
Omental VAT adipocyte hypoplasia in GDM-obese participants. (A) Representative images of H&E staining of NGT-nonobese, NGT-obese, GDM-nonobese, and GDM-obese omental VAT. Comparison of (B) adipocyte count (per 20× field image) (C) adipocyte size (µm^2^) and (D) fibrosis (% of image area) between NGT-nonobese (*n* = 9), NGT-obese (*n* = 3), GDM-nonobese (*n* = 4), and GDM-obese (*n* = 12) participants. (E) Representative images of IGFBP-3, IGFBP-4, and GAPDH protein expression in omental VAT from NGT-nonobese (*n* = 5), NGT-obese (*n* = 3), GDM-nonobese (*n* = 3), and GDM-obese (*n* = 5) samples. Densitometric analysis was used to quantify expression of (F) IGFBP-3 and (G) IGFBP-4 in study groups, with expression normalized to GAPDH. (H) Serum triglycerides (mM), (I) plasma IGF-1 (ng/mL) and (J) serum PAPPA levels (ng/mL) in NGT-nonobese (*n* = 13), NGT-obese (*n* = 7), GDM-nonobese (*n* = 7), and GDM-obese (*n* = 14) participants. Multiple linear regression was used to evaluate dependent variables between study groups, adjusted for maternal age. Significance levels are shown as: * *P* < .05, *** *P* < .001.

To evaluate adipocyte morphology and tissue fibrosis, biopsies of omental VAT were H&E stained (Fig. 1A). Participants in the GDM-obese category had significantly lower adipocyte counts per 20× image relative to NGT-nonobese participants, after adjusting for maternal age (*P* = .006) (Fig. 1B). Diagnosed treatment (diet-only, metformin, or insulin) had no significant effect on adipocyte count in GDM participants. There was no significant relationship between participant study groups and omental VAT adipocyte area, fibrosis, or IGFBP-3 and IGFBP-4 expression, serum triglycerides, plasma IGF-1, or serum PAPPA (Fig. 1C-1J).

### Dysfunctional Insulin Signaling in Omental VAT in GDM-Obese

**Figure 2.**
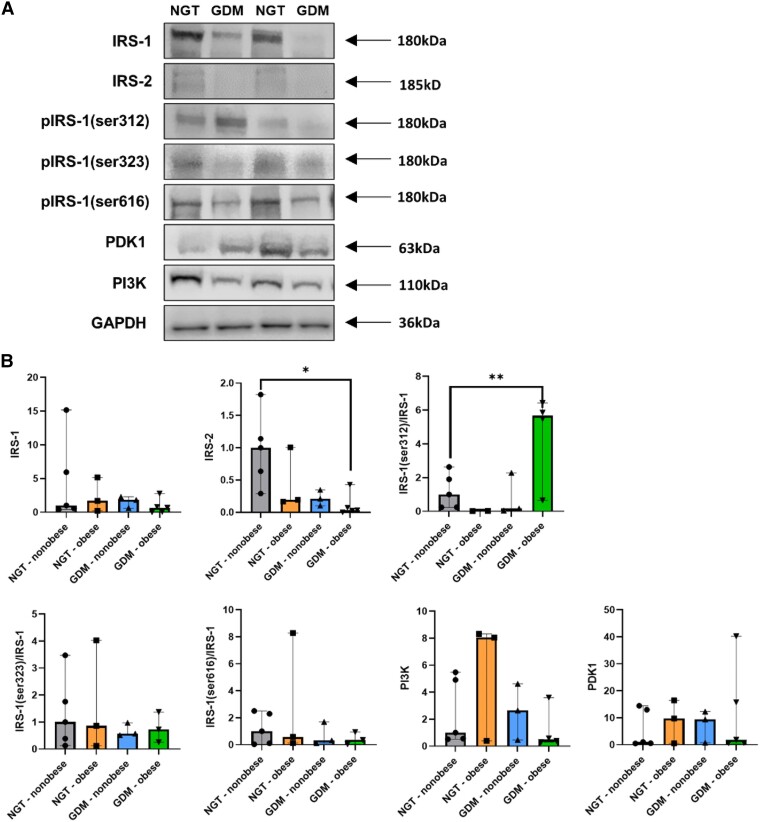
Modified insulin signaling in omental VAT of obese GDM participants. (A) Representative images of IRS-1, IRS-2, phospho-IRS-1(ser312), phospho-IRS-1(ser323), phospho-IRS-1(ser616), PDK1, PI3K, and GAPDH protein expression in omental VAT from NGT-nonobese (*n* = 5), NGT-obese (*n* = 3), GDM-nonobese (*n* = 3), and GDM-obese (*n* = 5) participants. (B) Densitometry analysis of total IRS-1, total IRS-2, phospho-IRS-1(ser312)/IRS-1, phospho-IRS-1(ser323)/IRS-1, phospho-IRS-1(ser616)/IRS-1, PDK1, and PI3K, with expression normalized to GAPDH. Multiple linear regression was used to compare relative protein levels with participant study groups, adjusted for maternal age, and IRS-2 and phospho-IRS-1(ser312)/IRS-1 levels with GDM treatment options, adjusted for maternal BMI and age. Significance levels are shown as: * *P* < .05, ** *P* < .01.

To elucidate if adipocyte hypoplasia in VAT alters insulin signaling in GDM, protein expression of members of the insulin signaling cascade were measured in omental VAT from participants with NGT and GDM (Fig. 2A).

Although there were no significant differences in protein expression of total IRS-1 in VAT between study groups, total IRS-2 was significantly decreased in the VAT of participants in the GDM-obese category relative to those in the NGT-nonobese category (*P* = .01). Furthermore, there was significantly increased phosphorylation of ser312 residue of IRS-1, relative to total IRS-1, in VAT of GDM-obese relative to NGT-nonobese participants (*P* = .006). We found no significant differences in the phosphorylation of IRS-1 serine residues ser323 and ser616 in VAT. Additionally, there was no statistically significant differences in the protein expression of PDK1 or PI3K in omental VAT between study groups (Fig. 2B). Diagnosed treatment (diet-only, metformin or insulin) had no significant effect on total IRS-2 expression or phosphorylation of ser312 residue of IRS-1 in omental VAT of GDM participants.

### Altered Adipokine Secretion in GDM Subgroups

**Figure 3.**
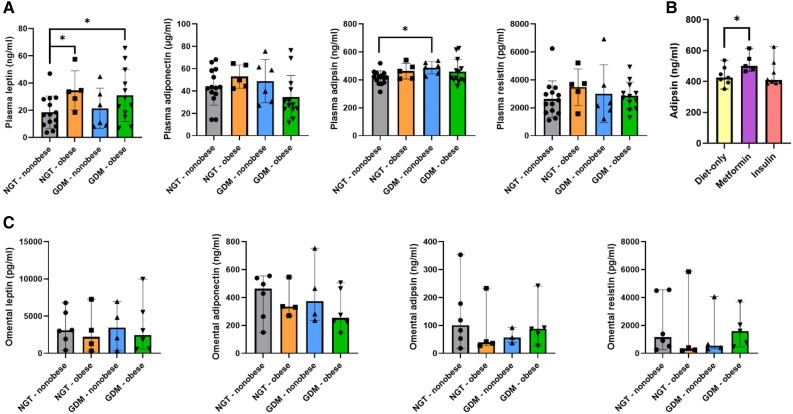
GDM subgroups have significantly altered circulating adipokine profiles. (A) Circulating levels of leptin, adiponectin, adipsin, and resistin were measured in fasting plasma samples from NGT-nonobese (*n* = 15), NGT-obese (*n* = 5), GDM-nonobese (*n* = 6), and GDM-obese (*n* = 14) participants. (B) Circulating levels of adipsin in GDM participants treated with diet-only (*n* = 7), metformin (*n* = 5), and insulin (*n* = 8). (C) Omental secretion of leptin, adiponectin, adipsin, and resistin was from NGT-nonobese (*n* = 6), NGT-obese (*n* = 4), GDM-nonobese (*n* = 4), and GDM-obese (*n* = 6) explant cultures. Multiple linear regression was used to compare adipokine levels with participant study groups, adjusted for maternal age, and plasma adipsin levels with GDM treatment options, adjusted for maternal BMI and age. Significance levels are shown as: * *P* < .05.

Four adipokines (leptin, adiponectin, resistin, and adipsin) were measured in fasting plasma samples from all study groups. Circulating plasma levels of leptin were significantly higher in both NGT-obese (*P* = .04) and GDM-obese (*P* = .02), relative to NGT-nonobese participants. Furthermore, plasma adipsin levels were significantly higher in GDM-nonobese relative to NGT-nonobese participants (*P* = .04). There were no significant differences in the circulating levels of adiponectin or resistin between study groups (Fig. 3A). After adjustment for maternal BMI and age, plasma adipsin levels were significantly elevated in metformin-treated GDM participants, relative to those treated with diet alone (*P* = .045) (Fig. 3B). No significant treatment effect (diet-only, metformin, or insulin) was seen on plasma leptin levels in GDM participants.

There was no significant difference between study groups in omental VAT production of leptin, adiponectin, adipsin, or resistin (Fig. 3C).

### Placental-Derived Mediators Do Not Directly Alter Visceral Adipocyte Function

**Figure 4.**
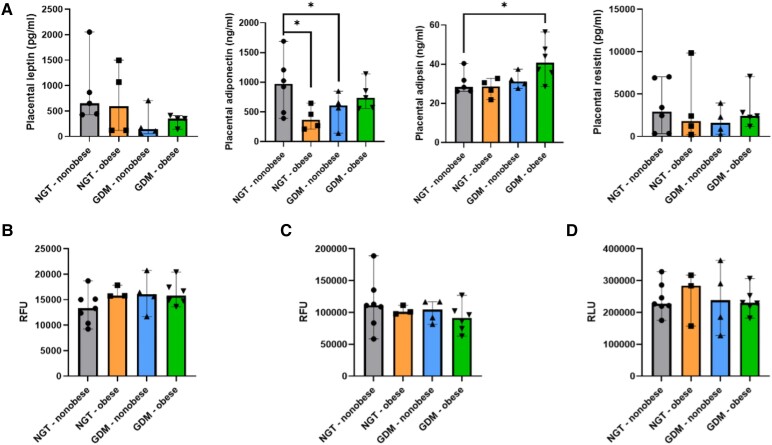
Placental-derived mediators do not directly impact adipocyte function. (A) Placental secretion of leptin, adiponectin, adipsin, and resistin was from NGT-nonobese (*n* = 6), NGT-obese (*n* = 4), GDM-nonobese (*n* = 4), and GDM-obese (*n* = 6) explant cultures. (B) Adipogenesis in differentiating preadipocytes, (C) intracellular lipid content in terminally differentiated mature adipocytes, and (D) glucose uptake in terminally differentiated mature adipocytes after treatment with NGT-nonobese (*n* = 7), NGT-obese (*n* = 3), GDM-nonobese (*n* = 4), and GDM-obese (*n* = 6) placental explant culture supernatant. Multiple linear regression was used to compare adipokine levels and assay outputs with participant study groups, adjusted for maternal age, and placental adiponectin and adipsin levels with GDM treatment options, adjusted for maternal BMI and age. Abbreviations: RFU, relative fluorescent units; RLU, relative luminescent units. Significance levels are shown as: * *P* < .05.

To investigate the potential of placental-derived adipokines to impact omental VAT functionality in GDM, leptin, adiponectin, resistin, and adipsin were measured in placental tissue explants. Compared to NGT-nonobese, there was a significant reduction in placental adiponectin secretion in both the NGT-obese (*P* = .01) and GDM-nonobese (*P* = .03) participants. There was a significant increase in placental adipsin release in GDM-obese relative to NGT-nonobese (*P* = .02). There were no significant differences in the placental production of either leptin or resistin between study groups (Fig. 4A). No significant treatment effect (diet-only, metformin, or insulin) was seen on placental adiponectin or adipsin levels in GDM participants.

To examine the effect of placental-derived mediators on adipocyte flexibility, a number of functional assays were performed. Initially, adipogenic differentiation was determined where preadipocytes cultured in a differentiation cocktail were treated with 5% placental explant culture supernatant from NGT (*n* = 10) and GDM (*n* = 10) participants during a 3-day differentiation protocol. Preadipocyte differentiation was characterized by increased lipid uptake. However, there was no significant difference in preadipocyte differentiation (relative fluorescent units [RFU]) between treatment groups (Fig. 4B).

We also investigated if placental signaling in GDM may alter adipocyte lipolysis. Intracellular lipid content was quantified in mature adipocytes, following 24-hour treatment with 5% NGT (*n* = 10) and GDM-ID (*n* = 10) placental explant supernatant. However, there were no significant differences in intracellular lipid content (relative fluorescent units) between treatment groups (Fig. 4C).

To investigate if placental mediators alter glucose uptake in mature adipocytes, glucose uptake was measured using the bioluminescent detection of 2-deoxyglucose-6-phosphate (2DG6P) in mature adipocytes following 24-hour treatment with 5% NGT (*n* = 10) and GDM (*n* = 10) placental explant supernatant. There were no significant differences in glucose uptake (relative luminescent units; RLU) between treatment groups (Fig. 4D).

## Discussion

Given the identified pathologic role of VAT in type 2 diabetes, we postulated that impaired adipocyte inflexibility in VAT would contribute to characterizing metabolic dysfunction in GDM. We classified NGT and GDM participants, according to their BMI, into either nonobese (<30 kg/m^2^) or obese (≥30 kg/m^2^) cohorts. In GDM-obese participants, we found significant adipocyte hypoplasia in omental VAT depots, relative to nonobese NGT samples. Insulin signal transduction in the VAT of GDM-obese participants was hindered by reduced total IRS-2 expression and elevated phosphorylation of the serine 312 residue of IRS-1. Furthermore, distinct profiles of adipokine dysregulation were found in study subgroups.

In this study, GDM participants had significant hyperinsulinemia and systemic insulin resistance, as determined using the HOMA-IR score, relative to NGT participants. It is widely recognized that hypertrophic adipocytes are insulin resistant and develop due to lipid overload and/or defective differentiation capacity (18). Adipocyte hypertrophy also inversely correlates with insulin sensitivity in nonobese individuals, suggesting that metabolic dysfunction, rather than obesity, promotes systemic insulin resistance (19). Hypertrophy occurs prior to hyperplasia in order to accommodate elevated requirements for fat storage. Hyperplasia subsequently acts to equilibrate local tissue metabolism and promote adipose tissue homeostasis. A deficiency in preadipocyte proliferation and/or differentiation can therefore predispose individuals to exaggerated metabolic dysfunction and insulin resistance (20, 21). GDM-obese participants had significantly lower adipocyte counts in their VAT depots compared to NGT-nonobese participants. This may represent an inherent defect in preadipocyte commitment to mature adipocytes with a resultant reduced capacity to accommodate appropriate lipid storage in GDM.

To interrogate potential molecular drivers of this VAT phenotype in GDM-obese, circulating markers of IGF activity were investigated. IGF-1 and IGF-2 are peptide growth factors that are primarily produced by the liver and regulate adipogenesis in adipose tissue (22). IGFBP family members bind available IGF-1 and thereby regulate IGF signaling by increasing its half-life and blocking receptor binding (14). However, we found no evidence of differential IGF-1 secretion or omental VAT IGFBP expression between NGT and GDM participants. To further dissect the possible mechanism behind omental hypoplasia in VAT depots in our GDM-obese cohort, we measured circulating levels of PAPPA, a protease that cleaves IGFBPs to increase the proportion of free, receptor-accessible IGF-1 (23). However, there were no significant differences between study groups in plasma PAPPA levels at term.

In order to elucidate if adipocyte dysfunction disrupts systemic glucose homeostasis, we interrogated the intracellular machinery regulating glucose metabolism via GLUT4-mediated glucose uptake. In this study, we identified increased phosphorylation at the ser312 residue of IRS-1 in GDM-obese participants only, impairing the ability of IRS-1 to activate downstream PI3K-dependent pathways. Additionally, total IRS-2 expression was significantly lower in VAT of GDM-obese participants relative to NGT-nonobese participants. Catalano et al reported significantly higher IRS-2 protein expression in subcutaneous adipose tissue in GDM participants compared with nonpregnant participants, but they did not observe a difference between NGT and GDM pregnancies (24). However, no studies to date have examined the omental expression of IRS-2 in GDM. In 3T3-L1 murine preadipocytes, knockdown of IRS-2 severely impaired insulin-stimulated AKT phosphorylation and glucose uptake and hindered preadipocyte differentiation (25). These results mirror our findings in GDM-obese participants, where we have shown both systemic and localized insulin resistance and reduced omental adipocyte count.

Higher circulating levels of leptin have been previously associated with obesity and type 2 diabetes, where neuroendocrine leptin resistance inhibits the ability of leptin to modulate food intake and body weight (26, 27). In this study, we observed obesity-related increases in leptin in both NGT-obese and GDM-obese participants. Elevated plasma adipsin levels were evident in GDM-nonobese, relative to NGT-nonobese participants. Although little is known about the biological role of adipsin in healthy pregnancy, it has been shown to have a beneficial role in maintaining beta cell function (28), and elevated circulating levels of adipsin have been reported previously in GDM pregnancies (29, 30). This may suggest that elevated production of adipsin is a homeostatic response to impaired insulin production in GDM-nonobese participants, promoting glycemic control in the insulin-resistant state of pregnancy. We found no significant dysregulation in omental VAT adipokine secretion.

Adipokines and/or their receptors are also expressed in placental tissue and contribute to the development of maternal insulin resistance (31). Adiponectin acts as an endogenous insulin sensitizer, promoting glucose metabolism while also exhibiting anti-inflammatory and anti-atherogenic properties (32). In our study, placental adiponectin secretion was significantly lower in NGT-obese and GDM-nonobese participants, compared to NGT-nonobese. Previous research has shown significantly reduced placental expression of adiponectin in GDM pregnancies, where placental adiponectin secretion is diminished by inflammatory cytokines such as IL-6, TNF-α, and IFN-γ. Furthermore, leptin treatment of healthy placental tissue significantly reduced adiponectin secretion, which may explain reduced placental adiponectin secretion in NGT-obese participants in this study, as this cohort also had elevated circulating leptin levels (33). Although no previous studies have examined the placental secretion of adipsin in GDM, higher circulating levels have been reported in women with GDM (29). Additionally, increased placental secretion of adipsin has been observed in pregnant obese women (34, 35). GDM-obese participants in this study had significantly elevated placental secretion of adipsin. As adipsin is the rate-limiting enzyme in the formation of acylation stimulating protein, a factor contributing to lipid storage and glucose uptake in adipose tissue, this may represent a regulatory pathway to help minimize the deleterious effects of GDM-induced hyperglycemia (34).

With our evidence of dysregulated placental adipokine production, we assessed the direct impact of NGT and GDM placental-derived mediators on visceral adipocytes. However, no significant impact was seen on adipogenesis, mature adipocyte lipid content, or adipocyte glucose uptake between study groups. These findings may indicate that adipocyte hypoplasia evident in GDM-obese VAT in this study is not mediated by placental-derived signals but may be related to a physiological deficiency in the commitment of preadipocytes toward a mature adipogenic lineage.

In this study, strict exclusion criteria ensured that only nulliparous women, without significant comorbidities or physiological adaptive changes from previous pregnancies, were included. Women were recruited on the day of cesarean delivery, with identical methods of sample collection and processing used for each participant. However, some limitations do exist. First, our study is predominantly cross-sectional in nature, eliminating the possibility of deriving causation from our findings. This research was carried out in Cork, Ireland, resulting in a largely White Irish participant group, thereby limiting the extrapolation of our findings to other ethnicities. This is of particular relevance to GDM as race-ethnicity and country of birth have a significant impact on the prevalence of this condition (36). This study shows that VAT adipocyte dysfunction may contribute to GDM and has identified potential molecular cues leading to metabolic inflexibility which warrant further examination in future longitudinal studies.

## Data Availability

The datasets generated and analyzed, and resources used, during the current study are available from the corresponding author upon reasonable request.

## Abbreviations

BMI: body mass index
FFA: free fatty acid
GDM: gestational diabetes mellitus
H&E: hematoxylin and eosin
HOMA-IR: homeostatic model assessment for insulin resistance
IGF-1: insulin-like growth factor 1
IGFBP: insulin-like growth factor binding protein
NGT: normal glucose tolerance
PAPPA: pregnancy associated plasma protein A
VAT: visceral adipose tissue

## References

[1] Ryan EA . Hormones and insulin resistance during pregnancy. Lancet. 2003;362(9398):1777‐1778. 14654313 10.1016/S0140-6736(03)14942-2

[2] Behboudi-Gandevani S, Amiri M, Bidhendi Yarandi R, Ramezani Tehrani F. The impact of diagnostic criteria for gestational diabetes on its prevalence: a systematic review and meta-analysis. Diabetol Metab Syndr. 2019;11:11. 30733833 10.1186/s13098-019-0406-1 PMC6359830

[3] Saeedi M, Cao Y, Fadl H, Gustafson H, Simmons D. Increasing prevalence of gestational diabetes mellitus when implementing the IADPSG criteria: a systematic review and meta-analysis. Diabetes Res Clin Pract. 2021;172:108642. 33359574 10.1016/j.diabres.2020.108642

[4] Paulo MS, Abdo NM, Bettencourt-Silva R, Al-Rifai RH. Gestational diabetes mellitus in Europe: a systematic review and meta-analysis of prevalence studies. Front Endocrinol (Lausanne). 2021;12:691033. 34956073 10.3389/fendo.2021.691033 PMC8698118

[5] Santomauro AT, Boden G, Silva ME, et al Overnight lowering of free fatty acids with Acipimox improves insulin resistance and glucose tolerance in obese diabetic and nondiabetic subjects. Diabetes. 1999;48(9):1836‐1841. 10480616 10.2337/diabetes.48.9.1836

[6] Sartipy P, Loskutoff DJ. Monocyte chemoattractant protein 1 in obesity and insulin resistance. Proc Natl Acad Sci U S A. 2003;100(12):7265‐7270. 12756299 10.1073/pnas.1133870100 PMC165864

[7] Fruhbeck G, Catalan V, Rodriguez A, Gomez-Ambrosi J. Adiponectin-leptin ratio: a promising index to estimate adipose tissue dysfunction. Relation with obesity-associated cardiometabolic risk. Adipocyte. 2018;7(1):57‐62. 29205099 10.1080/21623945.2017.1402151 PMC5915018

[8] Alharbi KK, Khan IA, Abotalib Z, Al-Hakeem MM. Insulin receptor substrate-1 (IRS-1) Gly927Arg: correlation with gestational diabetes mellitus in Saudi women. Biomed Res Int. 2014;2014:146495. 24695443 10.1155/2014/146495 PMC3948357

[9] Barbour LA, McCurdy CE, Hernandez TL, Friedman JE. Chronically increased S6K1 is associated with impaired IRS1 signaling in skeletal muscle of GDM women with impaired glucose tolerance postpartum. J Clin Endocrinol Metab. 2011;96(5):1431‐1441. 21289241 10.1210/jc.2010-2116 PMC3085211

[10] Catalano PM . Trying to understand gestational diabetes. Diabet Med. 2014;31(3):273‐281. 24341419 10.1111/dme.12381 PMC4178541

[11] Suarez-Cuenca JA, De La Pena-Sosa G, De La Vega-Moreno K, et al Enlarged adipocytes from subcutaneous vs. visceral adipose tissue differentially contribute to metabolic dysfunction and atherogenic risk of patients with obesity. Sci Rep. 2021;11(1):1831. 33469087 10.1038/s41598-021-81289-2 PMC7815822

[12] Trivett C, Lees ZJ, Freeman DJ. Adipose tissue function in healthy pregnancy, gestational diabetes mellitus and pre-eclampsia. Eur J Clin Nutr. 2021;75(12):1745‐1756. 34131300 10.1038/s41430-021-00948-9 PMC8636251

[13] Rojas-Rodriguez R, Lifshitz LM, Bellve KD, et al Human adipose tissue expansion in pregnancy is impaired in gestational diabetes mellitus. Diabetologia. 2015;58(9):2106‐2114. 26067361 10.1007/s00125-015-3662-0 PMC4526585

[14] Allard JB, Duan C. IGF-binding proteins: why do they exist and why are there so many? Front Endocrinol (Lausanne). 2018;9:117. 29686648 10.3389/fendo.2018.00117 PMC5900387

[15] Barbour LA, McCurdy CE, Hernandez TL, Kirwan JP, Catalano PM, Friedman JE. Cellular mechanisms for insulin resistance in normal pregnancy and gestational diabetes. Diabetes Care. 2007;30(Suppl 2):S112‐S119. 17596458 10.2337/dc07-s202

[16] Napso T, Yong HEJ, Lopez-Tello J, Sferruzzi-Perri AN. The role of placental hormones in mediating maternal adaptations to support pregnancy and lactation. Front Physiol. 2018;9:1091. 30174608 10.3389/fphys.2018.01091 PMC6108594

[17] Osman OS, Selway JL, Kepczynska MA, et al A novel automated image analysis method for accurate adipocyte quantification. Adipocyte. 2013;2(3):160‐164. 23991362 10.4161/adip.24652 PMC3756104

[18] Kim JI, Huh JY, Sohn JH, et al Lipid-overloaded enlarged adipocytes provoke insulin resistance independent of inflammation. Mol Cell Biol. 2015;35(10):1686‐1699. 25733684 10.1128/MCB.01321-14 PMC4405637

[19] Hammarstedt A, Graham TE, Kahn BB. Adipose tissue dysregulation and reduced insulin sensitivity in non-obese individuals with enlarged abdominal adipose cells. Diabetol Metab Syndr. 2012;4(1):42. 22992414 10.1186/1758-5996-4-42 PMC3523053

[20] Muir LA, Neeley CK, Meyer KA, et al Adipose tissue fibrosis, hypertrophy, and hyperplasia: correlations with diabetes in human obesity. Obesity (Silver Spring). 2016;24(3):597‐605. 26916240 10.1002/oby.21377 PMC4920141

[21] Sorisky A, Molgat AS, Gagnon A. Macrophage-induced adipose tissue dysfunction and the preadipocyte: should I stay (and differentiate) or should I go? Adv Nutr. 2013;4(1):67‐75. 23319125 10.3945/an.112.003020 PMC3648741

[22] Denley A, Cosgrove LJ, Booker GW, Wallace JC, Forbes BE. Molecular interactions of the IGF system. Cytokine Growth Factor Rev. 2005;16(4-5):421‐439. 15936977 10.1016/j.cytogfr.2005.04.004

[23] Byun D, Mohan S, Yoo M, Sexton C, Baylink DJ, Qin X. Pregnancy-associated plasma protein-A accounts for the insulin-like growth factor (IGF)-binding protein-4 (IGFBP-4) proteolytic activity in human pregnancy serum and enhances the mitogenic activity of IGF by degrading IGFBP-4 in vitro. J Clin Endocrinol Metab. 2001;86(2):847‐854. 11158056 10.1210/jcem.86.2.7223

[24] Catalano PM, Nizielski SE, Shao J, Preston L, Qiao L, Friedman JE. Downregulated IRS-1 and PPARgamma in obese women with gestational diabetes: relationship to FFA during pregnancy. Am J Physiol Endocrinol Metab. 2002;282(3):E522‐E533. 11832353 10.1152/ajpendo.00124.2001

[25] Groeneveld MP, Brierley GV, Rocha NM, Siddle K, Semple RK. Acute knockdown of the insulin receptor or its substrates Irs1 and 2 in 3T3-L1 adipocytes suppresses adiponectin production. Sci Rep. 2016;6:21105. 26888756 10.1038/srep21105 PMC4758029

[26] Kelesidis T, Kelesidis I, Chou S, Mantzoros CS. Narrative review: the role of leptin in human physiology: emerging clinical applications. Ann Intern Med. 2010;152(2):93‐100. 20083828 10.1059/0003-4819-152-2-201001190-00008 PMC2829242

[27] Liu W, Zhou X, Li Y, et al Serum leptin, resistin, and adiponectin levels in obese and non-obese patients with newly diagnosed type 2 diabetes mellitus: a population-based study. Medicine (Baltimore). 2020;99(6):e19052. 32028423 10.1097/MD.0000000000019052 PMC7015632

[28] Lo JC, Ljubicic S, Leibiger B, et al Adipsin is an adipokine that improves beta cell function in diabetes. Cell. 2014;158(1):41‐53. 24995977 10.1016/j.cell.2014.06.005 PMC4128197

[29] Saucedo R, Valencia J, Moreno-Gonzalez LE, et al Maternal serum adipokines and inflammatory markers at late gestation and newborn weight in mothers with and without gestational diabetes mellitus. Ginekol Pol. 2022;93(2):126‐133. 10.5603/GP.a2021.0083 33914332

[30] Vejrazkova D, Lischkova O, Vankova M, et al Distinct response of fat and gastrointestinal tissue to glucose in gestational diabetes mellitus and polycystic ovary syndrome. Physiol Res. 2017;66(2):283‐292. 27982680 10.33549/physiolres.933366

[31] D'Ippolito S, Tersigni C, Scambia G, Di Simone N. Adipokines, an adipose tissue and placental product with biological functions during pregnancy. Biofactors. 2012;38(1):14‐23. 22287297 10.1002/biof.201

[32] Ziemke F, Mantzoros CS. Adiponectin in insulin resistance: lessons from translational research. Am J Clin Nutr. 2010;91(1):258S‐261S. 19906806 10.3945/ajcn.2009.28449C PMC2793112

[33] Chen J, Tan B, Karteris E, et al Secretion of adiponectin by human placenta: differential modulation of adiponectin and its receptors by cytokines. Diabetologia. 2006;49(6):1292‐1302. 16570162 10.1007/s00125-006-0194-7

[34] Sivakumar K, Bari MF, Adaikalakoteswari A, et al Elevated fetal adipsin/acylation-stimulating protein (ASP) in obese pregnancy: novel placental secretion via Hofbauer cells. J Clin Endocrinol Metab. 2013;98(10):4113‐4122. 23956345 10.1210/jc.2012-4293 PMC3790615

[35] Lekva T, Lyle R, Roland MC, et al Gene expression in term placentas is regulated more by spinal or epidural anesthesia than by late-onset preeclampsia or gestational diabetes mellitus. Sci Rep. 2016;6:29715. 27405415 10.1038/srep29715 PMC4942618

[36] Hedderson MM, Darbinian JA, Ferrara A. Disparities in the risk of gestational diabetes by race-ethnicity and country of birth. Paediatr Perinat Epidemiol. 2010;24(5):441‐448. 20670225 10.1111/j.1365-3016.2010.01140.x PMC4180530

